# A pilot study to examine the association between immigration experience and health profiles in older Chinese Americans

**DOI:** 10.1002/alz70860_107596

**Published:** 2025-12-23

**Authors:** Shreya Upendra, Lorene Leung, Li Zhou, Jessica Spat‐Lemus, Ankit Parekh, Mahmood Mohamed, Drew Melancon, Amy Aloysi, Judith A. Neugroschl, Yiyu Cao, Linghsi Liu, Hiuyu Au, Joanne Lim, Clara Li

**Affiliations:** ^1^ Icahn School of Medicine at Mount Sinai, New York, NY, USA; ^2^ Montclair State University, Montclair, NJ, USA

## Abstract

**Background:**

Although prior research has examined the influence of immigration on physical and mental health conditions across diverse racial and ethnic groups, studies focusing specifically on Chinese Americans remain limited. Our pilot study, therefore, aims to explore this association to help identify risk and protective factors for Alzheimer's Disease and Alzheimer's Disease‐Related Dementias (AD/ADRD) among Chinese Americans who immigrated to the U.S.

**Method:**

88 Chinese American older adults (primary language: 48.9% Mandarin, 51.1% Cantonese), with either normal cognition (67%) or mild cognitive impairment (33%), were recruited from Mount Sinai's Alzheimer's Disease Research Center (ADRC). Immigration data, including age at immigration, duration (i.e., length of time in the U.S.), and acculturation, were collected via questionnaire and/or clinical interviews. Health outcomes were measured using Forms A5 and D1 of the National Alzheimer's Coordinating Center Uniform Data Set Version 3.0 (NACC UDS 3.0). Regression models assessed the relationship between immigration experience and health outcomes, controlling for age, gender, education, and socioeconomic status.

**Result:**

Participants, with a mean age of 71.2 years (70.5% female), had a median of 12 years of education, immigrated at median age of 34.5, and reported being in the U.S. for a median of 38 years. Common comorbidities included hypertension (60.2%), hyperlipidemia (78.2%), diabetes (30.7%), and insomnia (41.4%). Earlier age at immigration and longer immigration duration were linked to a higher risk of cerebrovascular disease (*p* = 0.033, *p* = 0.049, respectively). A higher degree of Chinese acculturation was associated with reduced likelihood of endorsing symptoms of anxiety and depression (*p* = 0.043, *p* = 0.02, respectively).

**Conclusion:**

This preliminary study suggests a plausible relationship between immigration experience and health outcomes in older Chinese Americans. Specifically, our pilot data suggested a higher risk of cerebrovascular disease for those who immigrated early in their life and a potential protective factor of cultural identity in mental health outcomes. Future studies should further examine this relationship in a larger sample to understand the implications of these factors in AD/ADRD risks and intervention.

